# Efficacy and Safety of Remimazolam Tosilate Combined With Esketamine for Analgesic Sedation in Mechanically Ventilated ICU Patients: A Single-Arm Clinical Study Protocol

**DOI:** 10.3389/fmed.2022.832105

**Published:** 2022-03-17

**Authors:** Xuan Song, Feng Wang, Ranran Dong, Kehan Zhu, Chunting Wang

**Affiliations:** ^1^Shandong Provincial Hospital Affiliated to Shandong First Medical University, Jinan, China; ^2^Dong E Hospital Affiliated to Shandong First Medical University, Liaocheng, China

**Keywords:** mechanical ventilation, intensive care unit, analgesics, sedation, remimazolam tosilate, esketamine

## Abstract

**Introduction:**

Patients in the intensive care unit (ICU) frequently experience increased heart rate, blood pressure, and respiration rate as a product of anxiety and restlessness about their condition and treatments. Analgesia and sedation commonly involve benzodiazepines or opioids that lead to respiratory suppression and other adverse reactions. Remimazolam tosilate is a short-acting GABA_A_ receptor agonist with reduced cardiovascular and respiratory inhibition compared to other commonly used benzodiazepines. Esketamine is a non-competitive N-methyl-D-aspartic acid (NMDA) receptor inhibitor that inhibits hyperalgesia and prolongs postoperative analgesia. It also reduces postoperative pain, delirium, and the use and acute tolerance of opioids. This study aims to assess the efficacy and safety of remimazolam tosilate combined with esketamine and sufentanil for sedation and analgesia in mechanically ventilated ICU patients.

**Methods and Analysis:**

This prospective, single-arm, single-center, open-label clinical trial will be conducted from January 2022 to December 2023. The study will include 200 adult patients (≥ 18 years) from Shandong Provincial Hospital (affiliated with Shandong First Medical University) who are mechanically ventilated and admitted to the ICU between 24 and 72 h from the time of ventilation and who are administered analgesia and sedatives. Patients will undergo arterial blood gas analysis before administration. Remimazolam tosilate (0.2 mg/kg) will be injected intravenously within 30 s, followed by continuous infusion at a rate of 0.1 to 0.3 mg/kg/h via micropump. Esketamine (0.25 mg/kg) will be injected intravenously and maintained at 0.15 mg/kg/h, while sufentanil will be maintained at the rate of 0.1 to 0.2 μg/kg/h. The primary study outcome is the overall time required to maintain sedation. Secondary outcomes will include the total dosage used to reach the target sedation level, total mechanical ventilation time, awakening time, length of hospital stay, and incidence of cardiorespiratory-related adverse events and delirium. Adverse events (AEs) will be reported regardless of their relationship to the experimental drugs. AEs associated with adverse drug reactions will be classified as “affirmative correlation,” “possible relevance,” and “unable to determine.” A paired *t*-test or Wilcoxon signed-rank test will be used to compare the changes of observed indexes before and after treatment. A *P* < 0.05 will be considered statistically significant.

**Ethics and Dissemination:**

This study was approved by the local ethics committee at Shandong Provincial Hospital affiliatied to Shandong First Medical University. The results of this trial will be disseminated in peer-reviewed journals and at scientific conferences.

**Trial Registration:**

The trial is registered at the Chinese Clinical Trial Registry: ChiCTR2100053106; date of registration: 2021-11-10.

## Introduction

Patients in the intensive care unit (ICU) frequently experience anxiety and restlessness related to concerns about their serious health condition (1) or disease prognosis and pain or discomfort associated with procedures such as intubation and mechanical ventilation. This state of stress can trigger increases in heart rate, blood pressure, oxygen consumption, and respiration rate, posing difficulty in clinical monitoring and treatment. Analgesia and sedation are effective in reducing metabolic rate and oxygen consumption in ICU patients, allowing them to adapt to an impaired oxygen transport state, minimizing further damage to the organs, and expediting the recovery of organ function (2–4). However, most commonly used analgesics and sedation treatments rely on liver and kidney metabolism, increasing the burden on the liver and kidney and increasing the risk for adverse reactions such as respiratory depression and hemodynamic instability, which is particularly dangerous among ICU patients (5).

The 2018 Chinese Adult ICU Analgesia and Sedation Treatment Guidelines (6) recommend the use of benzodiazepines and propofol for sedation in ICU patients (balanced propofol sedation, BPS). However, despite the rapid onset of action and short recovery time, propofol carries the risk of adverse effects such as temporary respiratory inhibition, hypotension, and bradycardia, particularly in patients with poor cardiac reserve function and low blood volume (7). For analgesia, the guidelines also recommend opioids such as sufentanil, which carries the risk for respiratory depression, decreased blood pressure, and reduced gastrointestinal peristalsis. Therefore, routine use of propofol combined with sufentanil for analgesia and sedation is limited in ICU patients.

Remimazolam tosilate is a new benzodiazepine sedative, which is active at the γ-aminobutyric acid subtype A (GABA_A_) receptor (8) and is used for intravenous anesthesia and anxiety relief. It is an ultra-short-acting GABA_A_ receptor agonist with a rapid effect, a controllable degree of cardiovascular inhibition, and negligible respiratory inhibition (9, 10). Additionally, esketamine, a pure right-handed enantiomer of ketamine, causes non-competitive inhibition of N-methyl-D-aspartic acid (NMDA) receptors, leading to neuroprotection, bronchiectasis, anti-hyperalgesia, and antiepileptic effects. Clinical studies have shown that low-dose esketamine can reduce postoperative pain, the dosage of postoperative analgesics, the incidence of delirium, and the use and acute tolerance of opioids; it also inhibits hyperalgesia and prolongs postoperative analgesia (1, 11, 12). The primary objective of this study is to investigate the efficacy and safety of remimazolam tosilate combined with esketamine and sufentanil for sedation in mechanically ventilated ICU patients.

## Methods and Analysis

### Study Design

This prospective, single-arm, single-center, open-label clinical trial will be conducted from January 2022 to December 2023. The primary sponsor of the trial is the Shandong Provincial Hospital, which is affiliated with the Shandong First Medical University. The design of the present study adheres to the Standard Protocol Items for Randomized Trials (SPIRIT) checklist (Supplementary Table 1).

### Population

**Figure 1:**
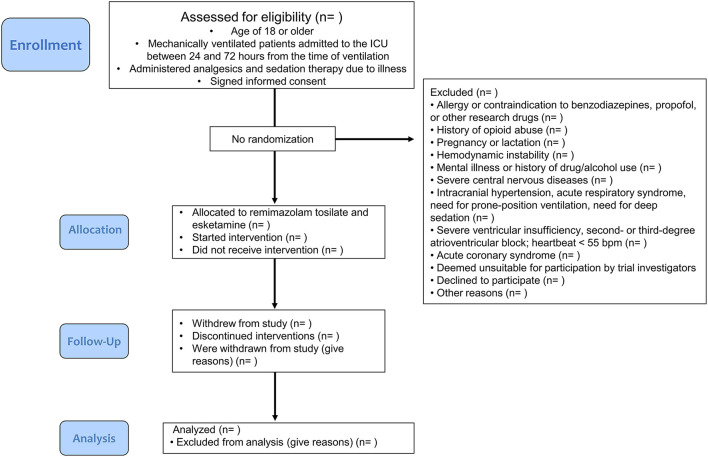
Flow chart of study design.

This study will include approximately 200 adult patients (≥18 years) at Shandong Provincial Hospital (affiliated with Shandong Medical University in the Shandong Province of China) who are mechanically ventilated and admitted to the ICU between 24 and 72 h from the time of ventilation, who are administered analgesia and sedation therapy due to illness, and who signed informed consent forms (Figure 1). Consent will be obtained by a study clinician who will explain the research procedures and participant rights to the patient or authorized surrogates. Because of the small, one-armed nature of this trial, the involvement of a data monitoring committee was not deemed to be necessary. Furthermore, we did not deem regular auditing to be necessary.

Exclusion criteria include the following: 1) allergy or contraindication to benzodiazepines, propofol, and other research drugs and their drug components; 2) history of opioid abuse; 3) pregnancy or lactation; 4) hemodynamic instability, often accompanied by a systolic blood pressure less than 90 mm Hg; 5) mental illness, history of drug use and/or alcohol abuse; 6) severe central nervous diseases (such as acute stroke, uncontrolled epilepsy, and severe dementia); 7) intracranial hypertension, acute respiratory distress syndrome, need for prone-position ventilation, and patients assessed by researchers as needing deep sedation; 8) severe ventricular insufficiency or second- or third-degree atrioventricular block; HR <55 beats/min; 9) acute coronary syndrome (such as unstable angina pectoris or acute myocardial infarction); 10) patients who are deemed unsuitable for participation in this trial by trial investigators.

Patients will also be eliminated from the study upon failure to comply with drug administration instructions. Patients can withdraw from the study at any time during the course of study for any reason, as long as the observation period specified in the program is completed. Patients will also be withdrawn from the study if the first weaning is not successful, requiring re-intubation. If the patient is withdrawn from the study due to allergic or adverse reactions or ineffective treatment, the investigators should take appropriate treatment measures based on the clinical condition of the patient. In the case of patients withdrawn from the study, investigators will make every effort to complete the evaluation to the best of their knowledge.

### Study Method

All patients will receive arterial blood gas analysis before administration. Remimazolam tosilate (Ruibeining^®^, Jiangsu Hengrui Pharmaceutical Co., Ltd.) will be injected intravenously (0.2 mg/kg) within 30 s, followed by continuous infusion of remimazolam tosilate at a rate of 0.1 to 0.3 mg/kg/h via micropump. Each adjustment will not exceed the sedation score of the patients monitored by 0.1 mg/kg/h (to be adjusted according to the sedation goal) until the target sedation level is reached (i.e., a Richmond Agitation-Sedation Scale [RASS] score of −2 to 0). Esketamine (Esther^®^, Jiangsu Hengrui Pharmaceutical Co., Ltd.) will be injected intravenously (0.25 mg/kg) and maintained at 0.15 mg/kg/h, while sufentanil will be maintained at the rate of 0.1 to 0.2 μg/kg/h.

During the period of sedation, patients with systolic blood pressure <80 mm Hg or >180 mm Hg, diastolic blood pressure <50 mm Hg or >100 mm Hg, and heart rate <40 beats/min or >120 beats/min will be given symptomatic treatment. During awakening, delirium and diagnostic delirium will be evaluated with the Confusion Assessment Method for the ICU (CAM-ICU). Droperidol (1 to 5 mg) will be injected intravenously to treat delirium, to be repeated at intervals of 10 to 20 min.

### Study Outcomes

#### Recording Observational Data

The arterial blood gas analysis parameters to be recorded prior to treatment will include pH, partial oxygen (PaO2), and partial carbon dioxide (PaCO2). Respiratory parameters will include respiration rate (RR) and oxygen saturation (SpO2). Circulatory parameters will include heart rate (HR), systolic blood pressure (SBP), and diastolic blood pressure (DBP) before drug administration, at the time of administration, and after administration (i.e., at 6, 12, 24, 48, and 72 h). Data quality will be ensured through a regular internal review process; research personnel will routinely review all databases for completeness and missing information.

The primary outcome is the percentage of time during which target sedation is maintained (RASS score of −2 to 0), expressed as a percentage of the overall study time. Secondary outcomes include the following: 1) total dosage of sedative and analgesic drugs to reach the target sedation level; 2) total mechanical ventilation time (from intubation to extubation), 3) RASS (Table 1) and Critical Care Pain Observation Tool (CPOT) (Table 2) scores every 4 h after administration, RASS and CPOT scores every 8 h after the first 24 h, and RASS and CPOT scores every 24 h after the first 48 h; 4) time of awakening (RASS score ≥0); 5) length of stay; 6) total dosage of all analgesics and sedatives; 7) incidence of bradycardia, hypotension, tachycardia, hypertension, delirium, and respiratory depression during the administration of analgesics and sedatives.

**Table 1:** Richmond agitation-sedation scale (RASS).

**Score**	**Term**	**Description**
+4	Combative	Overtly combative or violent; immediate danger to staff
+3	Very agitated	Pulls on or removes tube(s) or catheter(s), or has aggressive behavior toward staff
+2	Agitated	Frequent non-purposeful movement or patient-ventilator dyssynchrony
+1	Restless	Anxious or apprehensive but movements not aggressive or vigorous
0	Alert and calm	
−1	Drowsy	Not fully alert, but has sustained (>10 seconds) awakening with eye contact to voice
−2	Light sedation	Briefly (<10 seconds) awakens with eye contact to voice
−3	Moderate sedation	Any movement (but no eye contact) to voice
−4	Deep sedation	No response to voice, but any movement to physical stimulation
−5	Unarousable	No response to voice or physical stimulation

**Table 2:** The critical-care pain observation tool (CPOT).

**Indicator**	**Description**	**Score (Total score 0–8)**
Facial expressions	No muscle tension observed	Relaxed, Neutral 0
	Presence of frowning, brow lowering, orbit tightening and levator contraction or any other change (e.g., opening eyes or tearing during nociceptive procedures)	Tense **1**
	All previous facial movements plus eyelid tightly closed (the patient may present with mouth open or biting the endotracheal tube)	Grimacing **2**
Body movements	Does not move at all (doesn't necessarily mean absence of pain) or normal position (movements not aimed toward the pain site or not made for the purpose of protection)	Absence of movements or normal position **0**
	Slow, cautious movements, touching or rubbing the pain site, seeking attention through movements	Protection **1**
	Pulling tube, attempting to sit up, moving limbs/thrashing, not following commands, striking at staff, trying to climb out of bed	Restlessness/Agitation **2**
Muscle tension	No resistance to passive movements	Relaxed **0**
	Resistance to passive movements	Tense, rigid **1**
	Strong resistance to passive movements or incapacity to complete them	Very tense or rigid **2**
Compliance with the ventilator (intubated patients) OR	Alarms not activated, easy ventilation	Tolerating ventilator or movement **0**
	Coughing, alarms may be activated but stop spontaneously	Coughing but tolerating **1**
	Asynchrony: blocking ventilation, alarms frequently activated	Fighting ventilator **2**
Vocalization (extubated patients)	Talking in normal tone or no sound	Talking in normal tone or no sound **0**
	Sighing, moaning	Sighing, moaning **1**
	Crying out, sobbing	Crying out, sobbing **2**

Bradycardia is defined as a heart rate less than 55 beats/min or more than 20% below baseline during analgesia and sedation therapy (baseline <69 beats/min). Hypotension is defined as systolic blood pressure ≤95 mm Hg or more than 20% below baseline during analgesia and sedation therapy (baseline <119 mm Hg). Tachycardia is defined as a heart rate >100 bpm or more than 20% above the baseline (baseline >83 bpm); hypertension is defined as a systolic blood pressure >160 mm Hg or an increase of more than 20% above baseline (baseline value >133 mm Hg). Delirium is defined as occurring when a patient displays positive conditions during assessment with the CAM-ICU (Table 3) during analgesic and sedation therapy. Respiratory inhibition is defined as the occurrence of a respiratory frequency <8 breaths/min and/or blood oxygen saturation <90% during analgesia and sedation therapy.

**Table 3:** The confusion assessment method for the intensive care unit (CAM-ICU).

**Features and dscriptions**	**Absent**	**Present**
**I. Acute onset or fluctuating course**		
A. Is there evidence of an acute change in mental status from the baseline?		
B. Or, did the abnormal behavior fluctuate during the past 24 h, that is, tend to come and go or increase and decrease in severity as evidenced by fluctuations on the Richmond Agitation Sedation Scale (RASS) or the Glasgow Coma Scale?		
**II. Inattention**		
Did the patient have difficulty focusing attention as evidenced by a score of less than 8 correct answers on either the visual or auditory components of the Attention Screening Examination (ASE)?		
**III. Disorganized thinking**		
Is there evidence of disorganized or incoherent thinking as evidenced by incorrect answers to three or more of the four questions and inability to follow the commands?		
Questions		
1. Will a stone float on water?		
2.Are there fish in the sea?		
3.Does one pound weigh more than two pounds?		
4.Can you use a hammer to pound a nail?		
Commands		
1.Are you having unclear thinking?		
2.Hold up this many fingers. (Examiner holds two fingers in front of the patient.)		
3.Now do the same thing with the other hand (without holding the two fingers in front of the patient).		
(If the patient is already extubated from the ventilator, determine whether the patient's thinking is disorganized or incoherent, such as rambling or irelevant conversation, unclear or llogical flow of ideas, or unpredictable switching from subject to subject.)		
**IV. Altered level of consciousness**		
Is the patient's level of consciousness anything other than alert, such as being viqilant or lethargic or in a stupor, or coma?		
Alert: spontaneously fully aware of environment and interacts appropriately		
Vigilant: hyperalert		
Lethargic: drowsy but easily aroused, unaware of some elements in the environment or not spontaneously interacting with the interviewer; becomes fully aware and appropriately interactive when prodded minimally		
Stupor: difficult to arouse, unaware of some or all elements in the environment or not spontaneously interacting with the interviewer; becomes incompletely aware when prodded strongly; can be aroused only by vigorous and repeated stimuli and as soon as the stimulus ceases, stuporous subject lapses back into unresponsive state		
Coma: unarousable, unaware of all elements in the environment with no spontaneous interaction or awareness of the intervewer so that the interview is impossible even with maximal prodding		
**Overall CAM-ICU Assessment (Features 1 and 2 and either Feature 3 or 4): Yes___No___**		

#### Safety Evaluation

Criteria for adjudicating adverse events (AEs) include all unexpected clinical manifestations that occur after the informed consent is signed. All AEs will be reported regardless of their relationship to the experimental drugs.

Clinicians will comprehensively adjudicate the relationship between AEs and the experimental drugs and evaluate the possible association between the AEs and experimental drugs according to the five-level classification of 1) “affirmative correlation”: Occurrence of the event conforms to the reasonable time order after the use of the drug, the event conforms to the known reaction type of the suspected drug, improves after withdrawal, and the event occurs again after repeated administration; 2) “possible relevance”: Occurrence of the event conforms to the reasonable time sequence after the use of the drugs, the event conforms to the known reaction type of the suspected drug, and the patient's clinical state or other treatment may produce the event; 3) “possible irrelevance”: Occurrence of the event does not conform to the reasonable time sequence after medication, the event does not conform to the known reaction type of the suspected drug, and the event may occur in the patient's clinical state or other treatment; 4) “certainty irrelevance”: Occurrence of the event does not conform to the reasonable time sequence after the use of the drug, the event does not conform to the known reaction type of the suspected drug, and the patient's clinical state or other treatment may produce the event. Events are eliminated after the disease is improved or other treatments are stopped, and events occur after the repeated use of other treatments; 5) “unable to determine”: No clear relationship between the occurrence of events and the time sequence after administration, which is similar to the known reaction type of the drug, and other drugs used at the same time may also cause corresponding events.

AEs considered as “affirmative correlation,” “possible relevance,” and “unable to determine” will be classified as adverse drug reactions.

#### Follow-Up

All adverse events (including adverse drug reactions) that are not completely resolved at the end of the treatment course will be monitored until a complete resolution or until a stable condition is achieved.

### Statistical Analysis

Data related to patient characteristics and outcomes will be tested for normality and presented as percentages for categorical variables or as the mean and standard deviation for continuous variables as appropriate. Our per-protocol analysis will exclude patients who deviated from the protocol. A paired *t*-test will be used to compare the changes of observed indexes before and after treatment. When data are not normally distributed, a non-parametric Wilcoxon signed-rank test will be used to compare the paired data. A *P* < 0.05 will be considered statistically significant. SPSS 20.0 software will be used for statistical calculations. As this is a small, preliminary study to gain basic insight into the combined use of remimazolam and esketamine, sample size will not be based on formal hypothesis testing or power calculations. Our study sample size of 200 is expected to provide sufficient data to summarize overall results. Data quality will be ensured through a regular internal review process. We will conduct routine reviews of all data for completeness and missing information.

## Discussion

This study is a prospective, single-arm, single-center clinical trial to examine the efficacy and safety of remimazolam tosilate combined with esketamine as a new sedation regimen for patients with ICU mechanical ventilation. The study will recruit 200 adult mechanically ventilated patients in the ICU who will be treated with remimazolam combined with esketamine for sedation and analgesia. The primary goal of the study is to assess the total time to maintain target sedation, with additional outcomes of interest related to the amount of sedative and analgesic medication to reach the targeted sedation level, as well as clinical outcomes such as total mechanical ventilation time, awakening time, length of hospital stay, and adverse events.

Sedation is an indispensable component of treatment for critically ill patients undergoing mechanical ventilation, but the optimal sedative agent remains unclear. Over 70% of ICU patients experience anxiety and restlessness due to their serious illnesses. They also experience pain and discomfort due to their condition and treatment procedures and fear of their disease prognosis and death (1). Anxiety and restlessness can complicate patient care and have adverse physiological effects such as increased heart rate, blood pressure, respiration rate, and oxygen consumption, which can stress organ function. Pharmacological sedation reduces the metabolic rate of patients, improves oxygen consumption and demand, and reduces the metabolic burden on various organs (2–4).

Propofol and benzodiazepines such as midazolam are the current standard for sedation therapy for ICU patients in China (6). Benzodiazepines are agonists of GABA receptors in the central nervous system, with anti-anxiety, amnesia, sedation, hypnotic, and anticonvulsant properties. Propofol is an intravenous sedative-hypnotic that takes effect quickly and for a short time. However, propofol significantly inhibits the cardiorespiratory systems and carries a risk for hypotension, hypoxia, and bradycardia, particularly among patients with poor cardiac reserve function and low blood volume (13, 14). Other possible adverse reactions associated with long-term use include hypertriglyceridemia, acute pancreatitis, striated muscle injury, and drug resistance (7). Dexmedetomidine, a high-affinity alpha-2 adrenergic receptor agonist, is an alternative sedative drug frequently used for sedation due to its reduced delirium effects (2). However, a recent randomized trial found no difference in mortality rates at 90 days compared to a control group treated with usual care and more adverse events in the dexmedetomidine group (15).

Remimazolam tosilate is an ultra-short-acting GABA_A_ receptor agonist with rapid effect, a controllable degree of cardiovascular inhibition, and negligible respiratory inhibition (9, 10). The advantages of remimazolam are related to its ability to be rapidly metabolized *in vivo* via non-specific esterases, independent of liver and kidney metabolism; it can thus be used in patients with hepatic and renal insufficiency (16). Additionally, rapid metabolism does not allow accumulation of remimazolam tosilate *in vivo* after long-term infusion and high dose, and its effect can be rapidly reversed by flumazenil, enhancing its safety profile. We believe that remimazolam has many clinical advantages, such as effective relief of anxiety and restlessness during treatment in the ICU, stable hemodynamics, rapid metabolism, a lack of accumulation, and its ability to effectively reduce the incidence of adverse reactions and improve patient satisfaction with treatment. Remimazolam is currently being tested in several clinical trials for sedation as an alternative to propofol or dexmedetomidine due to its mild inhibitory effect on the cardiorespiratory systems (17–22).

Opioids such as sufentanil are routinely selected for analgesia in the ICU. Opioid use has many limitations and can lead to respiratory depression, decreased blood pressure, and reduced gastrointestinal peristalsis, especially in the elderly. Esketamine, a pure right-handed enantiomer of ketamine, results in neuroprotection, bronchiectasis, anti-hyperalgesia, and antiepileptic effects by non-competitive inhibition of NMDA receptors. Many clinical studies have shown that the use of low-dose esketamine can reduce the acute tolerance of opioids, postoperative pain, the dosage of postoperative analgesics, and the incidence of delirium. It can also inhibit hyperalgesia and prolong the time of postoperative analgesia (1, 11, 12). We believe that the combination of remimazolam and esketamine will provide safe and reversible sedation and analgesia in mechanically ventilated patients in the ICU.

## Conclusions

This study aims to assess remimazolam combined with esketamine as a sedative drug regimen for clinical application in mechanically ventilated ICU patients. Remimazolam tosilate has many potential clinical advantages, such as effective relief of anxiety and restlessness during ICU treatment, stable hemodynamics, rapid metabolism, a lack of accumulation, and reduced adverse reactions related to liver and kidney metabolism. When combined with esketamine for analgesia, the use of opioids may be reduced, thereby reducing the incidence of adverse reactions and improving patient satisfaction with treatment.

## Ethics Statement

The studies involving human participants were reviewed and approved by Ethics Committee of Shandong Provincial Hospital Affiliated to Shandong First Medical University. The patients/participants provided their written informed consent to participate in this study.

## Author Contributions

CW and XS planned the study. XS performed the design of the study. XS and FW drafted the manuscript. CW critically revised the manuscript. All authors contributed to the design and development of the trial, read, and approved the final manuscript.

## Funding

This study was funded by SHDMA (Shandong Provincial Medical Association, No.YXH2020ZX032). This funding source had no role in the design of this study and will not have any role during its execution, analyses, interpretation of the data, or decision to submit results.

## Conflict of Interest

The authors declare that the research was conducted in the absence of any commercial or financial relationships that could be construed as a potential conflict of interest.

## Publisher's Note

All claims expressed in this article are solely those of the authors and do not necessarily represent those of their affiliated organizations, or those of the publisher, the editors and the reviewers. Any product that may be evaluated in this article, or claim that may be made by its manufacturer, is not guaranteed or endorsed by the publisher.

## Abbreviations

CAM-ICU: Confusion Assessment Method for the ICU
CPOT: Critical Care Pain Observation Tool
RASS: Richmond Agitation-Sedation Scale.
